# Impaired Tight Junctions in Atopic Dermatitis Skin and in a Skin-Equivalent Model Treated with Interleukin-17

**DOI:** 10.1371/journal.pone.0161759

**Published:** 2016-09-02

**Authors:** Takuo Yuki, Megumi Tobiishi, Ayumi Kusaka-Kikushima, Yukiko Ota, Yoshiki Tokura

**Affiliations:** 1 Biological Science Research, Kao Corporation, Odawara, Japan; 2 Department of Dermatology, Hamamatsu University School of Medicine, Hamamatsu, Japan; Emory University School of Medicine, UNITED STATES

## Abstract

Tight junction (TJ) dysfunction in the *stratum granulosum* leads to aberrant barrier function of the *stratum corneum* (SC) in the epidermis. However, it is unclear whether TJs are perturbed in atopic dermatitis (AD), a representative aberrant SC-related skin disease, and whether some factors related to AD pathogenesis induce TJ dysfunction. To address these issues, we investigated the alterations of TJs in AD skin and the effects of Th2 and Th17 cytokines on TJs in a skin-equivalent model. The levels of TJ proteins were determined in the epidermis of nonlesional and lesional skin sites of AD. Western blot and immunohistochemical analyses revealed that the levels of zonula occludens 1 were decreased in the nonlesional sites of AD, and the levels of zonula occludens 1 and claudin-1 were decreased in the lesional sites relative to the levels in skin from healthy subjects. Next, we examined the effects of interleukin (IL)-4, tumor necrosis factor-α, IL-17, and IL-22 on the TJ barrier in a skin-equivalent model. Only IL-17 impaired the TJ barrier. Furthermore, we observed a defect in filaggrin monomer degradation in the IL-17–treated skin model. Thus, TJs are dysfunctional in AD, at least partly, due to the effect of IL-17, which may result in an aberrant SC barrier.

## Introduction

Tight junctions (TJs) mediate one mode of cell-to-cell adhesion in the sheets of cells forming the epithelium and endothelium [1]. TJs comprise a network of sealing strands and act as a primary barrier to the diffusion of solutes through the intercellular space. Each strand is formed by a row of transmembrane proteins. Occludin and claudins are the major transmembrane proteins in TJs, and zonula occludens (ZO) form cytosolic scaffolds that regulate the assembly of tight junctions [2].

The epidermis forming the outermost layer of the human body is an epithelial tissue classified as stratified squamous epithelium. The main function of the epidermis is to provide a protective barrier against water loss and the penetration of infectious agents and allergens. The *stratum corneum* (SC) is largely responsible for this vital barrier function [3]. The TJ barrier is present beneath the SC barrier, and its constitutive proteins such as occludin, claudin-1, claudin-4, and ZO-1 are colocalized in the granular layer [4,5]. When an intercellular tracer is injected into the epidermis from the dermal side, it diffuses through most of the paracellular space but is blocked at positions where constitutive TJ proteins are coassembled [5,6]. These results show that TJs are present in the granular layer of the epidermis in mice and humans.

Several recent studies have attempted to determine the purpose of TJs in the epidermis. When the SC is disrupted, the dendrites of Langerhans cells usually present beneath the TJs penetrate the TJs and take up external antigens [7]. Langerhans cells elicit humoral immunity to antigens that have yet to rupture the epidermal barrier and thereby confer preemptive immunity against potentially pathogenic skin microbes [8]. Infiltration of pathogens into the epidermis has been shown to immediately enhance TJ function via Toll-like receptor signaling [9,10]. This finding indicates that dynamically controlled TJs are fundamental for preventing further invasion of pathogens and maintaining the cutaneous barrier homeostasis [9,10] and that TJ function changes in response to the external environment. Our group previously demonstrated an important role of TJs in the SC-based permeability barrier. In skin-equivalent models treated with a glutathione-S-transferase fusion protein with the C-terminal half of *Clostridium perfringens* enterotoxin, TJ impairment led to an aberrant SC barrier by affecting the processing of profilaggrin [11]. The same effects were observed in claudin-1 knockout mice [12]. Thus, TJs have crucial roles in the formation and function of the SC, which suggests that SC homeostasis cannot be maintained in diseases with TJ dysfunction. The abnormal localization of claudin-1 is present in patients with psoriasis and atopic dermatitis (AD) [13–15] and may therefore contribute to the vulnerability of the SC barrier.

The pathophysiology of AD is complex and involves abnormalities of barrier and immune functions. Loss-of-function mutations in the gene encoding filaggrin contribute to barrier perturbation [16], and filaggrin expression is significantly reduced in patients with AD even without filaggrin mutations [17]. In a study using filaggrin-null mice, filaggrin was shown to have a critical role in the SC integrity and the epicutaneous sensitization, which are important factors in early-phase AD [18]. However, 10% of the human population harbors filaggrin mutations, although far fewer people develop AD [19,20], which suggests that some factors other than filaggrin are deeply involved in the barrier dysfunction.

AD is a Th2-polarized disease, and common extrinsic AD patients express high levels of Th2 cytokines such as interleukin (IL)-4, IL-5, and IL-13 [21]. In addition, these patients exhibit high frequencies of circulating IL-4^+^ or IL-5^+^ Th2 cells, although the frequency of circulating interferon (IFN)-γ^+^ Th1 cells is higher in intrinsic than extrinsic AD [22]. However, a recent study of lesional skin showed that increased activation of all inflammatory axes, including Th2 cells, occurred in patients with intrinsic AD [23], which suggests an important role of Th2 cells in the development of both types of AD lesions. Furthermore, Th17 cells that produce IL-17A (IL-17 here) and IL-22 have a crucial role in AD pathogenesis. Th17 cells are increased in the peripheral blood of patients with AD, and Th17 cells infiltrate in acute skin lesions to a greater extent than they do chronic lesions [24]. In the lesional skin, positive correlations between Th17-related molecules and severity scores have been observed in intrinsic AD, whereas extrinsic AD has shown positive correlations between severity scores and Th2 cytokine (IL-4 and IL-5) levels [23].

It is possible that SC homeostasis cannot be maintained in skin disorders with TJ dysfunction [11]. An immunohistochemical analysis detected abnormal localization of claudin-1 in AD [13], although the quantitative changes in the levels of TJ proteins remain unknown. In this study, we performed a quantitative analysis of the formation of TJ constitutive proteins to identify the alterations of TJs in AD. Furthermore, the effects of Th2, Th17, and inflammatory cytokines on TJs were analyzed to clarify the mechanisms underlying TJ dysfunction associated with AD immunopathology.

## Results

### Decreased syntheses of ZO-1 and claudin-4 in nonlesional sites (NLS) and of ZO-1 and claudin-1 in lesional sites (LS) of AD skin

A TJ protein, occludin, was localized at cell-cell contact sites of the granular layer [4–6,24]. Three other TJ proteins, ZO-1, claudin-1, and claudin-4 were occurred from the basal to the granular layer but were assembled into the occludin-containing sites [4–6,24]. Tracer experiments revealed that intercellular tracer diffused through intercellular space from the basal to the granular layer, but the diffusion was stopped at the occludin localized sites where claudin-1 and -4 proteins were coassembled [5,6]. These results demonstrated that functionally maturated TJs that can prevent intercellular diffusion of solutes appear in the granular layer where occludin and claudin-1 and -4 were assembled.

**Fig 1:**
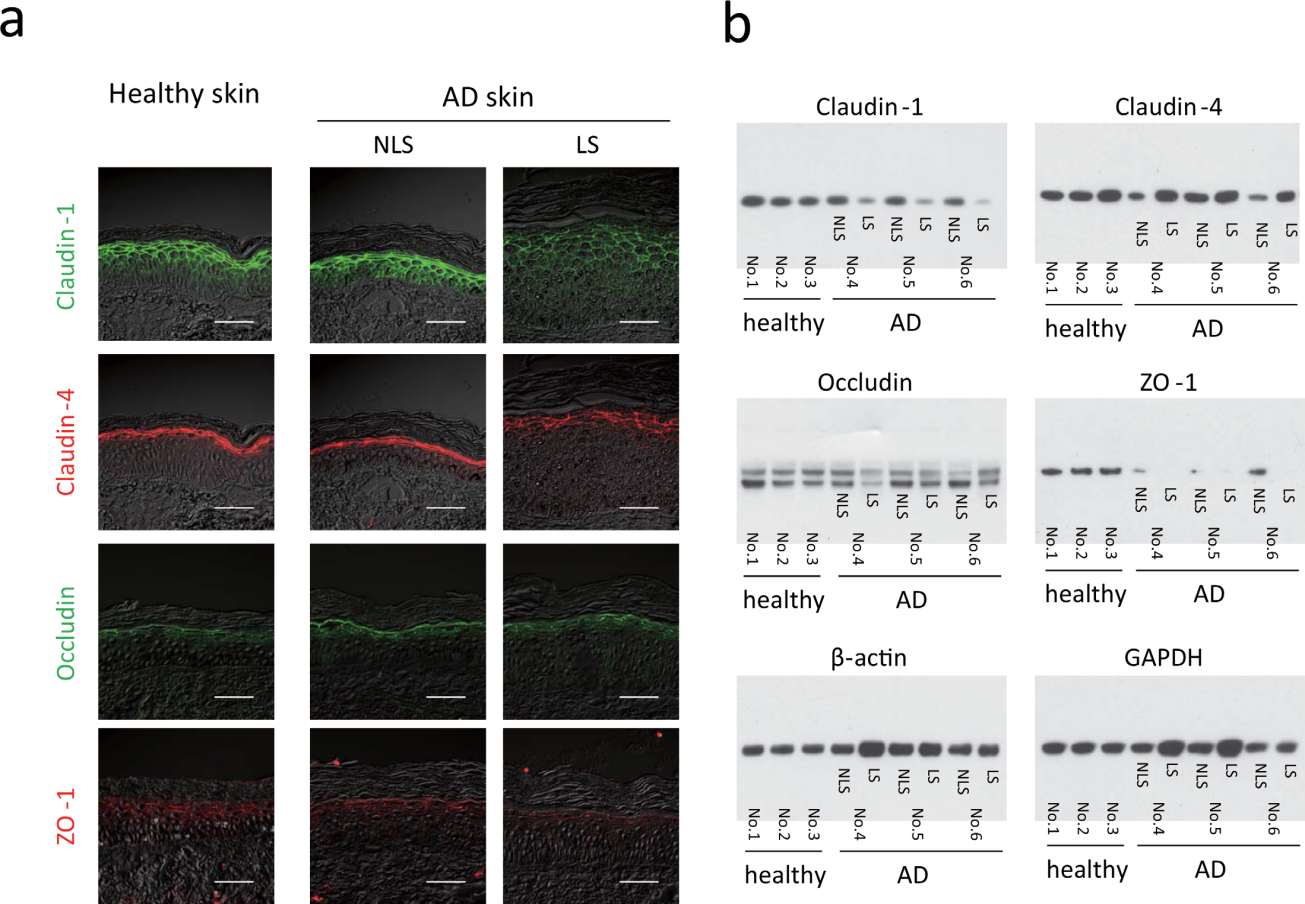
Reduced levels of zonula occludens 1 (ZO-1), claudin-1, and claudin-4 proteins in the epidermis of patients with atopic dermatitis (AD). Skin biopsies were taken from healthy subjects (Nos. 1–3) and patients with AD (Nos. 4, 5, 6). Nonlesional sites and lesional sites were biopsied in all patients with AD. (a) Double-immunostaining for claudin-1 [fluorescein isothiocyanate (FITC), green], claudin-4 (Cy3, red), occludin (FITC, green), and ZO-1 (Cy3, red). Scale bars = 50 μm. (b) Western blot analysis of claudin-1, claudin-4, ZO-1, occludin, β-actin and GAPDH syntheses.

To examine TJ barrier function in the epidermis of AD, TJ protein levels were quantified in the epidermal tissues of three patients with AD and three normal subjects. Skin biopsies were taken from AD NLS and LS and subjected to immunohistochemistry and western blotting. Representative immunolabeling of claudin-1, claudin-4, occludin, and ZO-1 (Fig 1A) in healthy skin detected the localization of claudin-1 protein from the basal to granular layers of the epidermis and that of claudin-4 in the granular layer, as reported previously [4,6]. Occludin and ZO-1 were observed at the cell–cell contact sites of the granular layer. In NLS of AD, claudin-1, claudin-4, occludin, and ZO-1 proteins occurred similarly to those in normal skin. In LS, however, the signal intensities of claudin-1 and ZO-1 were markedly reduced (Fig 1A).

Western blot analysis of three normal skin (Nos. 1–3) and three AD skin specimens (Nos. 4–6) confirmed significantly decreased claudin-1 protein production in LS relative to productions in NLS and normal skin (Fig 1B). The levels of claudin-4 were lower in the NLS of Nos. 4 and 6 than in normal skin samples, although LS produced claudin-4 protein at a level comparable to that in normal skin. ZO-1 protein was synthesized at very low levels in all three samples of LS and at moderately low levels in NLS relative to those in normal skin. A significant reduction in occludin synthesis was not detected in the NLS or LS. The significantly reduced ZO-1 protein synthesis in the NLS and LS, as well as that of claudin-1 in the LS, suggests that the TJ barrier is perturbed in the NLS and LS of AD skin. In fact, the TJ barrier was impaired by siRNA ZO-1 knockdown in the skin-equivalent model (S1 Fig).

### Perturbation of TJ barrier function by IL-17 in a skin-equivalent model

**Fig 2:**
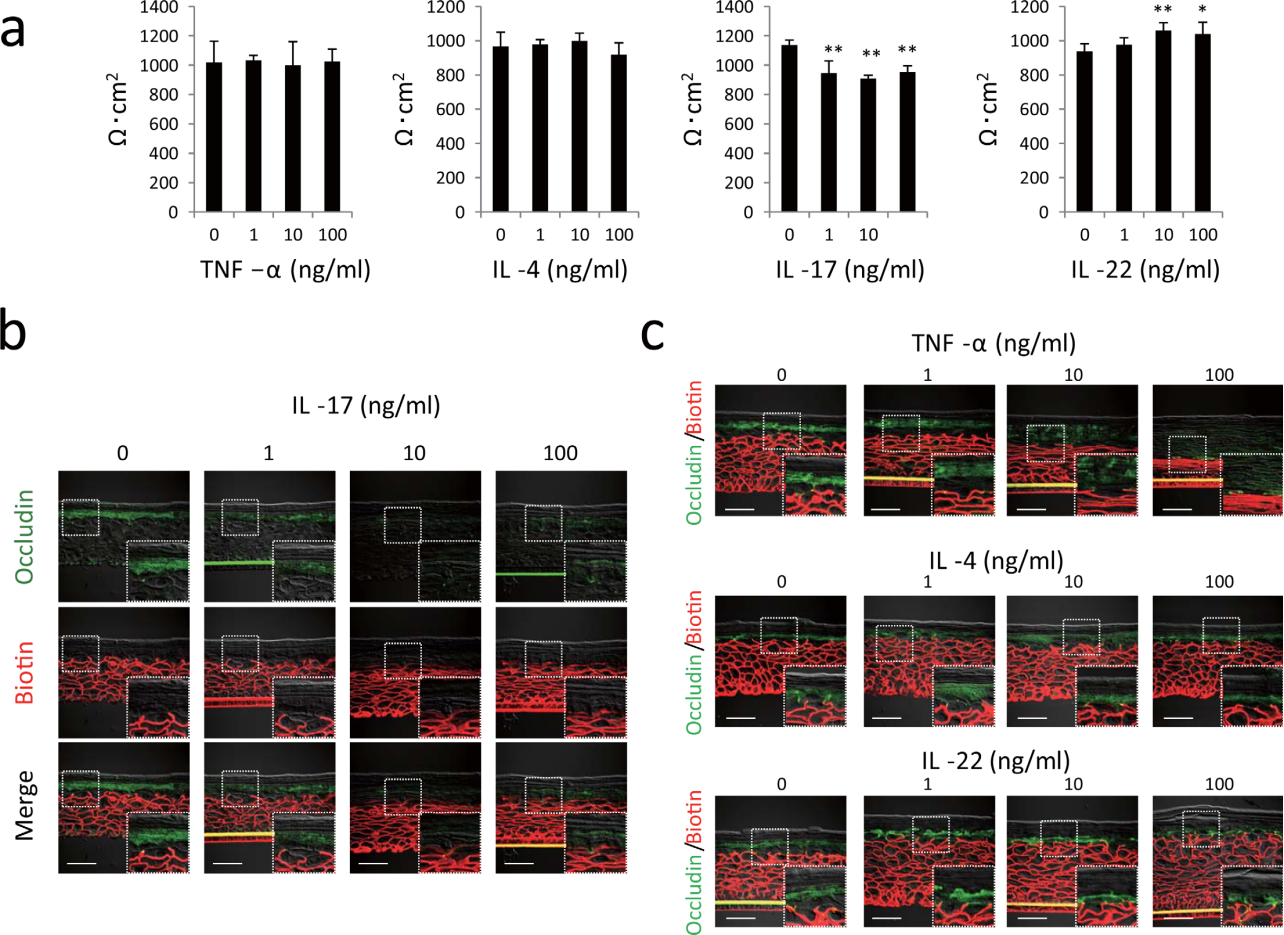
IL-17 impairs the tight junction (TJ) barrier in monolayer cultures of keratinocytes and in a skin-equivalent model. (a) Monolayer cultures of human keratinocytes were incubated with TNF-α, IL-4, IL-17, and IL-22, and the transepithelial electric resistance was then measured. Results are shown as the mean ± standard deviation. Results of a representative experiment are shown. *P < 0.05, **P < 0.01. (b) Human skin-equivalent model specimens were incubated with IL-17 and subjected to a TJ permeability assay by using sulfo-NHS-LC-Biotin (Texas Red; red) and concomitantly stained with occludin (fluorescein isothiocyanate; green). Sulfo-NHS-LC-biotin is identified beneath the sites where occludin is localized. (c) Human skin-equivalent model specimens were incubated with TNF-α, IL-4, and IL-22 and then subjected to the TJ permeability assay. Scale bars = 50 μm.

TJ barrier dysfunction may be related to the immunological abnormalities of AD. Th2 and Th17 cells and their cytokines are involved in the pathophysiology of the acute skin lesion of AD [25,26]. To test whether these cytokines affected the TJ barrier, we evaluated the effects of IL-4 (Th2 cytokine), IL-17, IL-22 (Th17 cytokines), and tumor necrosis factor (TNF)-α (proinflammatory cytokine) on the TJ barrier. Hence, normal human epidermal keratinocytes were cultured with TNF-α, IL-4, IL-17, or IL-22 for 2 days, and transepithelial electric resistance (TER) was measured as an index of the TJ barrier. IL-17 concentrations as low as 1 ng/ml significantly decreased the TER, whereas 10 ng/ml of IL-22 increased the TER (Fig 2A). TNF-α and IL-4 generated no observable effects.

A skin-equivalent model was subjected to a TJ tracer assay to evaluate the effect of each cytokine on the TJ barrier. The skin-model specimens were incubated with sulfo-NHS-LC-biotin as a paracellular tracer from the dermal side [6,11]. Immunofluorescent detection of occludin was performed after incubation (Fig 2B). Occludin protein was localized in the granular layer of an untreated control skin model (0 ng/ml IL-17). The injected intercellular tracer (biotin) diffused through the intercellular space from the basal to lower granular layers, but the diffusion was blocked in the area where occludin was localized (inset). In the skin-model specimen exposed to 10 and 100 ng/ml of IL-17, the intercellular tracer reached the upper granular layer unrestricted in the area where occludin was localized. TNF-α, IL-4, and IL-22 had no effect on the TJ barrier because the diffusion of the tracer at any concentration stopped along with occludin localization (Fig 2C). Thus, IL-17 attenuated the TJ barrier in cultured keratinocytes and in the skin-equivalent model.

### Reduction of ZO-1, claudin-1, and claudin-4 synthesis by IL-17 in the skin-equivalent model

**Fig 3:**
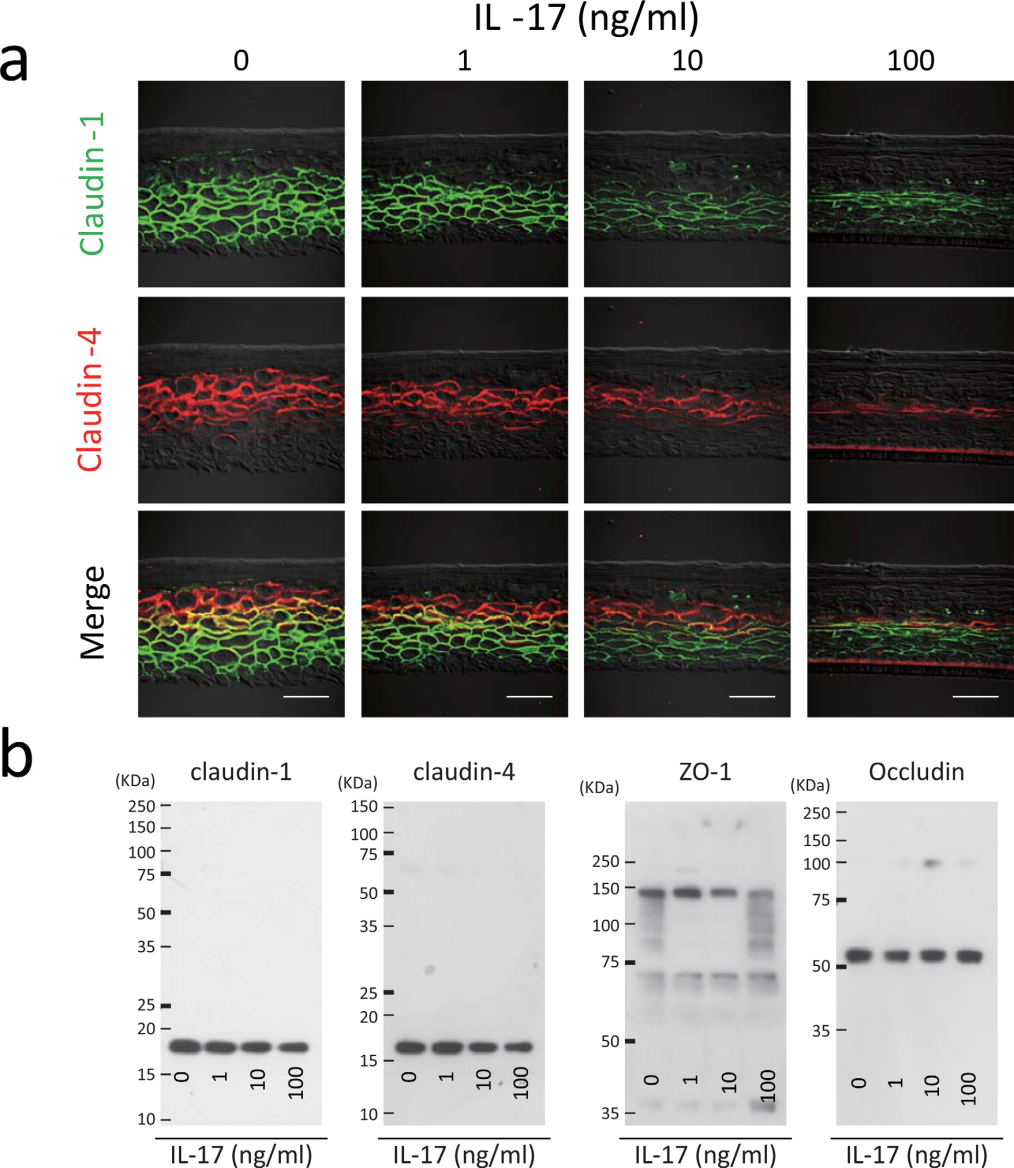
IL-17 decreases zona occludens 1 (ZO-1), claudin-1, and claudin-4 synthesis in a skin-equivalent model. (a) Double-immunostaining of claudin-1 (fluorescein isothiocyanate; green) and claudin-4 (Cy3, red) was performed in skin-equivalent model specimens incubated with IL-17. Scale bars = 50 μm. (b) Western blot analysis of the synthesis of claudin-1, claudin-4, ZO-1, and occludin in the skin-equivalent model incubated with IL-17.

Skin-equivalent model specimens were constructed in the presence of IL-17 and TJ proteins were analyzed by using immunohistochemistry and western blotting. The results of double-immunolabeling of claudin-1 and claudin-4 are depicted in Fig 3A. In the untreated skin model (0 ng/ml IL-17), claudin-1protein was localized from the basal to the lower granular layers, and claudin-4 protein was occurred from the spinous to the upper granular layers. Both proteins were coassembled in the lower granular layer, where the TJ presumably forms. In contrast, skin specimens treated with IL-17 exhibited profound decreases in the signals of claudin-1 and claudin-4 in a dose-dependent manner without any evidence of their colocalization.

Western blotting was performed to assess the amounts of claudin-1, claudin-4, ZO-1, and occludin as TJ constituents. Incubation with IL-17 at 10 or 100 ng/ml reduced the synthesis of claudin-1, claudin-4, and ZO-1proteins, but not occludin (Fig 3B). Thus, claudin-1 or claudin-4 was not detected in the lower granular layer when the skin specimen was constructed in the presence of IL-17. Moreover, the synthesis of ZO-1, claudin-1, and claudin-4 proteins were downregulated by IL-17.

These results indicated that downregulation of ZO-1, claudin-1, and claudin-4 proteins by IL-17 attenuated the TJ barrier in a skin-equivalent model.

### Delayed filaggrin processing by IL-17 in the skin-equivalent model

**Fig 4:**
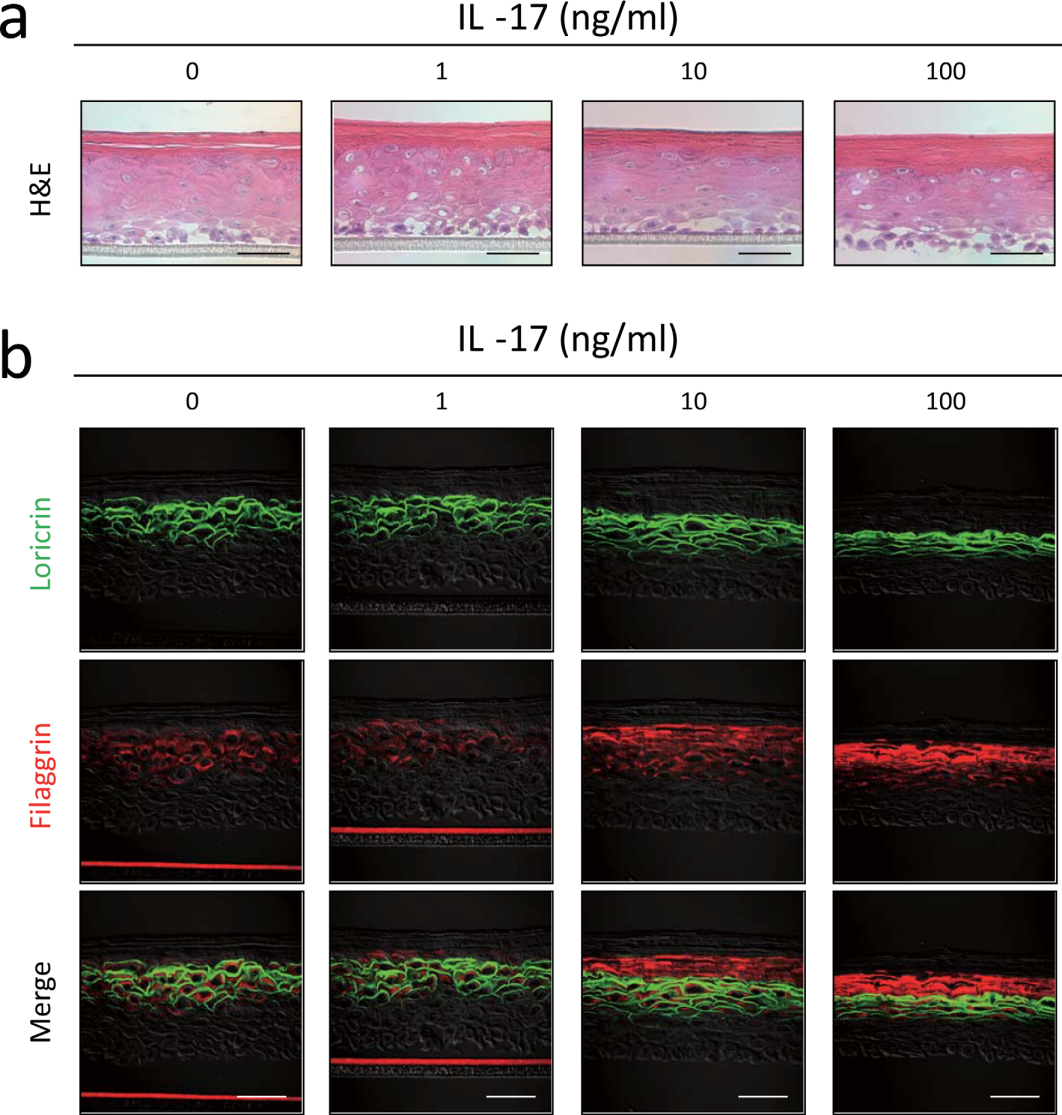
IL-17 alters filaggrin localization in skin-equivalent model. (a) Hematoxylin and eosin staining of the skin-equivalent model incubated with or without varying concentrations of IL-17. (b) Double-immunostaining of loricrin (fluorescein isothiocyanate, green) and filaggrin (RRX, red). Skin-model specimens were incubated with or without varying concentrations of IL-17. Scale bars = 50 μm.

Impairment of the TJ barrier leads to SC-barrier dysfunction associated with inhibition of filaggrin processing [11]. We examined whether IL-17 affected filaggrin processing in the skin-equivalent model in association with TJ dysfunction. Skin-equivalent model specimens were constructed in the presence or absence of IL-17, and the levels of profilaggrin gene expression, filaggrin protein synthesis, and filaggrin degradation substances, especially amino acids, were examined. Histological analysis of a skin-model specimen treated with 100 ng/ml IL-17 revealed marked thickening of the SC and relative thinning of the epidermis relative to those of the control skin (Fig 4A). In double-immunolabeling of the untreated skin specimen (Fig 4A; 0 ng/ml IL-17), loricrin protein was localized in the plasma membrane of the granular layer cells, and filaggrin protein was in their cytoplasm. Both proteins appeared in the granular layer and disappeared in the SC. The number of loricrin-bearing layers was decreased with reduced epidermal thickness by 10 or 100 ng/ml IL-17. In contrast, the filaggrin signal was increased by IL-17 treatment. The merged image shows that filaggrin was present above the loricrin-localized layers, indicating the presence of filaggrin in the SC.

**Fig 5:**
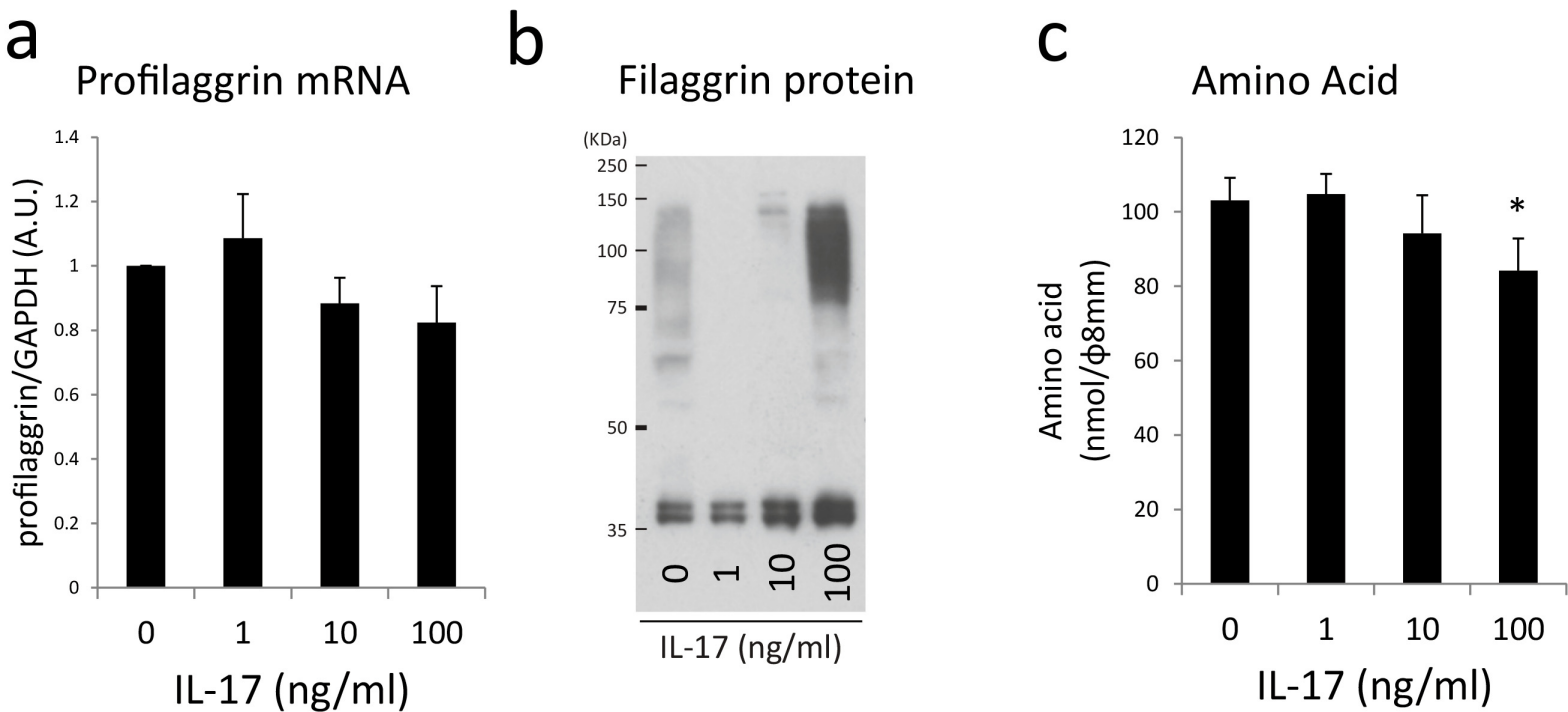
IL-17 alters profilaggrin processing in skin-equivalent model. (a) qRT–PCR analysis of filaggrin mRNA expression. (b) Western blot analysis of filaggrin synthesis.(c) Amino acid content of the SC. Amino acids extracted from SC were measured using the OPA method. *P < 0.05.

On the basis of these findings, degradation of filaggrin protein to amino acids may be impaired in IL-17-treated skin. Therefore, profilaggrin and filaggrin expressions were analyzed by using real-time reverse transcription polymerase chain reaction (qRT–PCR) and western blotting. qRT–PCR revealed a marginal decrease of profilaggrin mRNA expression in response to IL-17 (Fig 5A). Western blotting revealed that 10 or 100 ng/ml IL-17 increased the levels of filaggrin monomer and that 100 ng/ml IL-17 increased the levels of filaggrin dimers and trimers (Fig 5B). Furthermore, we observed that the quantities of amino acids extracted from the SC were decreased by 100 ng/ml IL-17 (Fig 5C), which suggested that aberrant degradation of filaggrin to amino acids leads to a reduction in the amino acids content in the SC.

## Discussion

We have previously demonstrated that defective TJ function leads to formation of an aberrant SC barrier, and therefore, hypothesized that an aberrant SC forms in skin diseases with TJ dysfunction [11]. The present study suggests that the TJ barrier function is impaired in AD skin, which may contribute to an aberrant SC barrier, a typical feature of AD. In the LS samples, the levels of claudin-1 and ZO-1 were decreased. Notably, the protein synthesis of ZO-1 in all of the NLS samples and claudin-4 in some of the NLS samples were similarly decreased. TJ barrier function was attenuated by silencing ZO-1 expression (S1 Fig) and is obstructed by claudin-4 [11,27]. The observation that claudin-1 mRNA expression is reduced in the NLS of AD [12] is inconsistent with our findings, but its decrease was observed in the LS, which might have been caused by the presence of subclinical changes in the NLS samples.

Considering that skin barrier perturbations and immunological abnormalities interact with each other, this raises the issue of whether AD-related cytokines affect TJs. Therefore, here we focused on Th2 and Th17 cytokines and found that incubation of the skin-equivalent model with IL-17 remarkably attenuated the TJ barrier. Th17 cells have been shown to infiltrate the skin lesions of AD, particularly during the acute phase of dermatitis [22], which suggested that IL-17 produced from Th17 cells decreases TJ function by inhibiting the synthesis of ZO-1, claudin-1, and claudin-4 proteins in AD. IL-17 has been shown to modify epithelial cells in the local milieu and stimulate keratinocytes to produce certain cytokines and chemokines [22]. Microarray analysis has revealed that IL-17 downregulates filaggrin gene expression and numerous genes that are important for cellular adhesion and possibly mediates epidermal barrier formation [28]. Moreover, occludin discontinuously occurs in the granular layer after IL-17 exposure [29]. Here we showed further that the levels of ZO-1, claudin-1, and claudin-4 were downregulated by Th17. Certain cytokines are overproduced locally as well as systemically in patients with AD, although their levels vary among patients. We observed that claudin-4 levels were decreased in the NLS of two of three patients, possibly because of the influence of other cytokines or individual variation, such as the extent of Th2 skewing. Notably, our finding provides important evidence for the difference in the effects of cytokines between the SC and TJ. Although profilaggrin synthesis has been shown to be reduced by the Th2 cytokines IL-4 and IL-13 [17], the synthesis of TJ-related proteins was inhibited by the Th17 cytokine IL-17.

Analysis of claudin-1 knockout mice has revealed that treatment with IL-17 caused thickening of the SC and abnormal filaggrin degradation as well as TJ dysfunction [5,12]. Claudin-1-deficient patients are known to suffer from neonatal ichthyosis-sclerosing cholangitis, a condition that manifests an ichthyosis skin phenotype [30]. The development of AD has not been reported to occur in patients with neonatal ichthyosis-sclerosing cholangitis; however, the development of AD in this disease remains unclear due to the paucity of patients. Notably, analysis of claudin-1 haplotype-tagging single nucleotide polymorphisms has revealed the association with AD in North American populations, and the abnormality in the epidermal TJ barrier may be linked to the development of AD [13].

The present study provides important information on the clinical significance of TJ in skin diseases, such as AD, because TJ’s function is impaired in patients with AD and results in an aberrant SC barrier. However, further studies are required to clarify the relationship between ZO-1, claudin-1, and claudin-4 and the pathogenesis of AD.

## Materials and Methods

### Subjects and sample preparation

Stephens and Associates (Carrollton, TX, USA) recruited AD patients with LS and NLS on their extremities along with healthy subjects as controls. AD was diagnosed according to the criteria published by a consensus conference held in the United States [31]. The characteristics of subjects are shown in S1 Table. Skin biopsies were obtained from the healthy subjects (Nos. 1–3) and patients with AD (Nos. 4–6) as well as LS and NLS only from the latter patients. The Institutional Review Board of IntegReview Ltd. (Austin, TX, USA) approved this study, and all subjects provided written informed consent.

### Cells and reagents

Normal human epidermal keratinocyte cells (Kurabo, Osaka, Japan) were cultured according to our previous report [10]. Briefly, normal human epidermal keratinocyte cells propagated in MCDB 153 medium (Nihon Pharmaceutical Co., Ltd., Tokyo, Japan) containing 0.1 mM Ca^2+^ with the following additives: 5 mg/L insulin, 180 μg/L hydrocortisone, 14.1 mg/L O-phosphorylethanolamine, 6.1 mg/L 2-aminoethanol, 100 ng/L epidermal growth factor, and 0.4% (vol/vol) bovine pituitary extract. The cells were trypsinized to prepare single cell suspensions, which were plated onto a Transwell filter (0.4-μm pores; Millipore, Bedford, MA, USA). When the cells reached confluency, they were transferred to medium containing 1.8 mM Ca^2+^. Two days later, TNF-α, IL-4, IL-17, and IL-22 were added, and the TJ permeability barrier was evaluated.

A human-skin-equivalent model was purchased from MatTek Corporation (Ashland, MA, USA) and used according to the manufacturer’s recommendations. Two days after the human-skin-equivalent model was received, TNF-α, IL-4, IL-17, and IL-22 were added into a medium prepared from the human-skin equivalents. Two days later, the TJ permeability barrier was evaluated.

TNF-α (Recombinant Human TNF-alpha, 210-TA-010), IL-4 (Recombinant Human IL-4, 204-IL-010/CF), IL-17 (Recombinant Human IL-17/IL-17A, 7955-IL-025), and IL-22 (782-IL-010) were purchased from R&D systems (Minneapolis, MN, USA).

### Histology, immunofluorescence staining, and microscopy

Human tissues from healthy subjects and AD patients were fixed in formaldehyde and embedded in paraffin. For routine histology, 5-μm sections were cut and placed on Superfrost Plus slides (Surgipath, Peterborough, UK). The sections were stained using standard methods with hematoxylin and eosin. Claudin-1 and claudin-4 were detected by citrate buffer (pH 6.0) antigen retrieval, followed by incubation with a rabbit polyclonal (pAb) anti-claudin-1 antibody (51–9000) (Invitrogen, Camarillo, CA, USA), a mouse monoclonal anti-claudin-4 antibody (32–9400) (Invitrogen), a mouse anti-ZO-1 monoclonal antibody (mAb) (33–9100) (Invitrogen) and a rat anti-occludin mAb (MOC37, a gift from Prof. Mikio Furuse, National Institute for Physiological Sciences, Japan). The avidity and specificity of the antibodies described here have already demonstrated [15,32–34]. Notably, occludin is highly concentrated at the TJs in most simple epithelial cells and the epidermis, so we used rat anti-occludin mAb for detecting the sites where TJs had formed [5,35–37].

Skin-equivalent tissues were placed in optimal cutting temperature compound (Sakura Finetek Japan, Tokyo, Japan) and frozen in a liquid nitrogen-cooled isopentane bath. Frozen tissue sections (5 μm) were fixed with 95% ethanol for 30 min, fixed with acetone for 2 min, blocked with 1% bovine serum albumin in phosphate-buffered saline (PBS), and immunolabeled by using the following primary antibodies: claudin-1 pAb, claudin-4 pAb (Invitrogen), and filaggrin mAb (clone 15C10) (Leica, Newcastle, UK), and loricrin pAb (Covance, Emeryville, CA, USA).

### Sample preparation and immunoblotting

Biopsied human skin was dipped in PBS at 70°C for 30 s, and the dermis was slipped off to obtain the epidermal sheet. Epidermal sheets were lysed in a buffer containing 200 mM Tris (pH 7.4), 2.0% sodium dodecyl sulfate, protease inhibitor cocktail (Roche, Mannheim, Germany), and phosphatase inhibitor cocktail (Roche, Mannheim, Germany). Then, we analyzed the protein levels of claudin-1, claudin-4, ZO-1, occludin, and filaggrin in the epidermis, according to our previous report [10]. The following primary antibodies were used: claudin-1 pAb, claudin-4 mAb, ZO-1 mAb, occludin pAb (all from Invitrogen), anti-loricrin pAb (Covance, Emeryville, CA, USA), and filaggrin mAb (clone 15C10; Leica, Newcastle, UK).

### qRT PCR

Total RNA was extracted by using an RNeasy kit (QIAGEN, Dusseldorf, Germany). Reverse transcription was performed by using a high-capacity cDNA Archive kit (Life Technologies). qPCR was performed by using the StepOne Real-Time PCR System (Life Technologies) according to the manufacturer’s instructions. The amount of mRNA was calculated from the cycle threshold values (experimentally determined number of PCR cycles required to achieve threshold fluorescence). Gene expression levels were standardized to those of the RPLP0 mRNA.

### TJ permeability assay

The TJ-associated barrier was evaluated in a human keratinocyte culture by using a Millicell–ERS epithelial volt meter to measure TER [24]. Alternatively, a TJ permeability assay was evaluated using EZ-Link Sulfo-NHS-LC-Biotin (MW 556.59; Pierce, Rockford, IL, USA) as a paracellular tracer in human skin models according to a published method [5,6,11]. Human skin models were incubated with 2 mg/mL sulfo-NHS-LC-Biotin in PBS containing 1 mM CaCl_2_ from the dermal side. After incubation for 30 min, the skin models were removed for preparation of frozen sections. Sections (5-μm thick) were fixed in 95% ethanol for 30 min, placed in 100% acetone for 2 min, soaked in 1% bovine serum albumin/PBS for 15 min, incubated with anti-occludin mAb for 1 h, washed three times with PBS, and incubated for 1 h with a mixture of FITC-conjugated anti-rat pAb (Jackson ImmunoResearch, West Grove, PA, USA) and streptavidin-Texas Red (Calbiochem, Farmstadt, Germany).

### Amino acid analysis

To extract soluble amino acids from the SC, 10 mM HCl was added to a skin-equivalent model completely formed over a Transwell filter, and the absence of leakage beneath the insert was confirmed. Thirty minutes later, the extracted amino acids were quantified by using O-phthalaldehyde, which is generally used as a fluorescent probe for amino acid analyzers. The detailed method for measuring the amino acid content has been previously published [38].

### Calculations and statistical analysis

All data were expressed as the mean ± standard deviation. Individual groups were compared by using the *t* test for pairwise comparisons. *P* < 0.05 was considered to indicate statistical significance for all comparisons.

## Supporting Information

S1 Fig The TJ barrier was impaired by ZO-1 knockdown in the skin-equivalent model. (PDF)

S2 Fig Western blotting for filaggrin in the skin equivalent model treated with IL-4, TNF-α, IL-17 and IL-22, respectively. (PDF)

S1 Table The details of recruited subjects. (PDF)

S2 Table The details of numeric data. (PDF)
