# Supplementary material for: Impaired Tight Junctions in Atopic Dermatitis Skin and in a Skin-Equivalent Model Treated with Interleukin-17
Source: PLoS One. 2016 Sep 2;11(9):e0161759. doi: 10.1371/journal.pone.0161759 (PMC5010286; doi:10.1371/journal.pone.0161759)

S1 Fig. The tight junction (TJ) barrier was impaired by ZO-1 knockdown in the skin-equivalent model.

(a) The skin-equivalent model (J-TEC, Kyoto, Japan) was used according to the manufacturer's instructions. Scramble siRNA or ZO-1 siRNA mixed with RNAiMAX was added to the Transwell filter and the keratinocytes were seeded onto the Transwell filter insert. After 1 day, the keratinocytes were exposed to air and stratification was induced. Ten days later, the expressions of ZO-1, occludin, and claudin-1 and -4 were measured by the real-time reverse transcription-polymerase chain reaction method. The skin model transfected with ZO-1 siRNA showed significantly decreased ZO-1 mRNA. In contrast, there were no effects on occludin or claudin-1 or -4.

(b) A TJ permeability assay was performed in the skin model transfected with ZO-1 siRNA. The injected intercellular tracer did not stop at the sites where occludin was expressed in this skin model transfected with ZO-1 siRNA

a

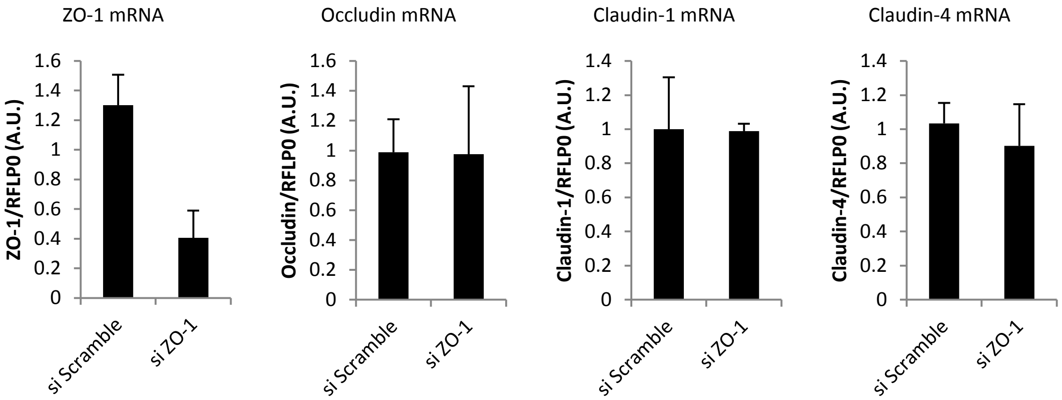

b

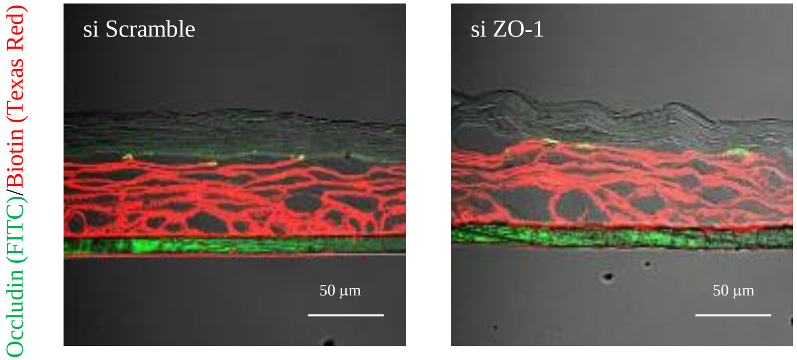

Supplement: S1 Fig — (PDF) [file pone.0161759.s001.pdf]
