# Supplementary material for: Impaired Tight Junctions in Atopic Dermatitis Skin and in a Skin-Equivalent Model Treated with Interleukin-17
Source: PLoS One. 2016 Sep 2;11(9):e0161759. doi: 10.1371/journal.pone.0161759 (PMC5010286; doi:10.1371/journal.pone.0161759)

**S2 Fig. Western blotting for filaggrin in the skin equivalent model treated with IL-4, TNFα, IL-17 and IL-22, respectively.**

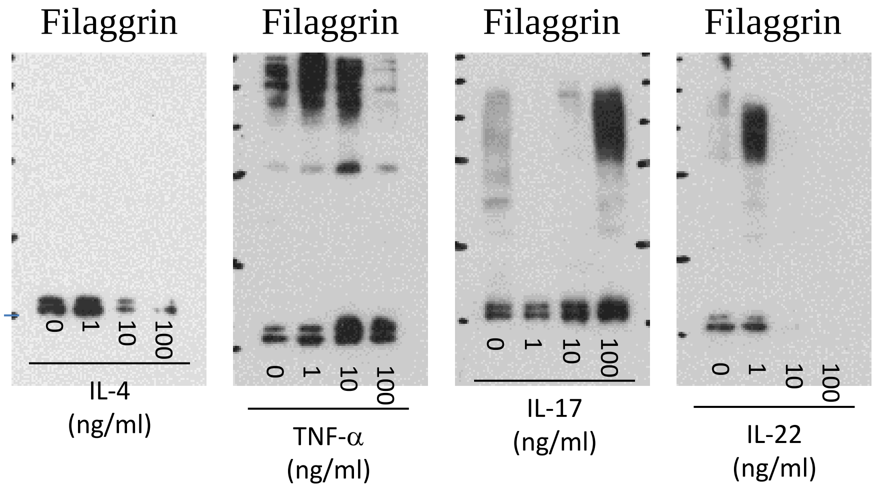

Supplement: S2 Fig — (PDF) [file pone.0161759.s002.pdf]
