# Supplementary material for: Impaired Tight Junctions in Atopic Dermatitis Skin and in a Skin-Equivalent Model Treated with Interleukin-17
Source: PLoS One. 2016 Sep 2;11(9):e0161759. doi: 10.1371/journal.pone.0161759 (PMC5010286; doi:10.1371/journal.pone.0161759)

S1 Table The details of recruited subjects

Subject No.	race	sex	age	Biopsied sites
001	Caucasian	Male	49	Posterior region of arm
002	Caucasian	Female	29	Posterior region of arm
003	Caucasian	Female	48	Posterior region of arm
004	Caucasian	Male	59	NLS, anterior region of arm; LS, leg
005	Caucasian	Female	61	NLS, anterior region of arm; LS, leg
006	Caucasian	Female	49	NLS, posterior region of arm/shoulder part<br>LS, posterior region of arm/elbow part

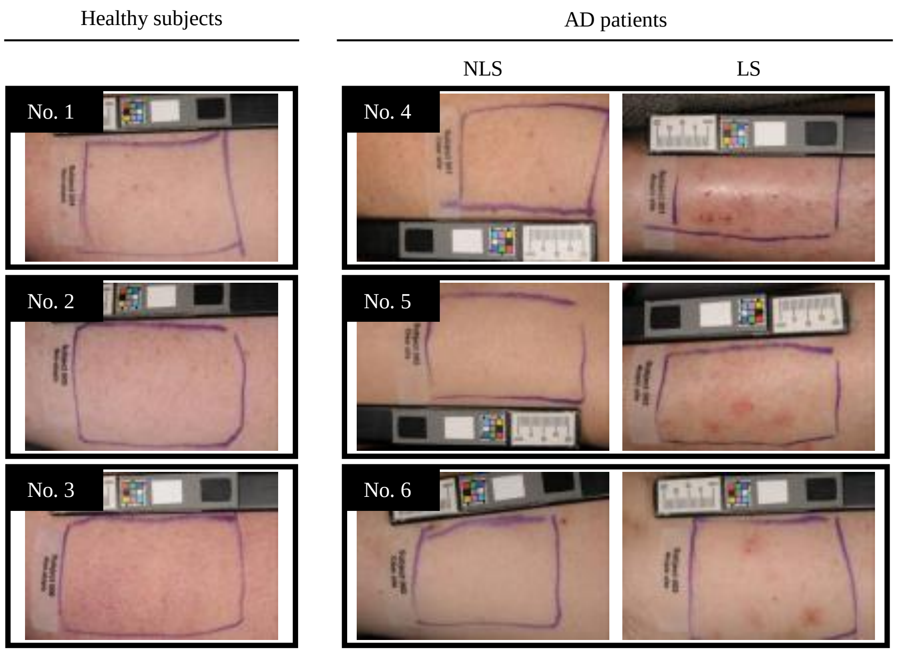

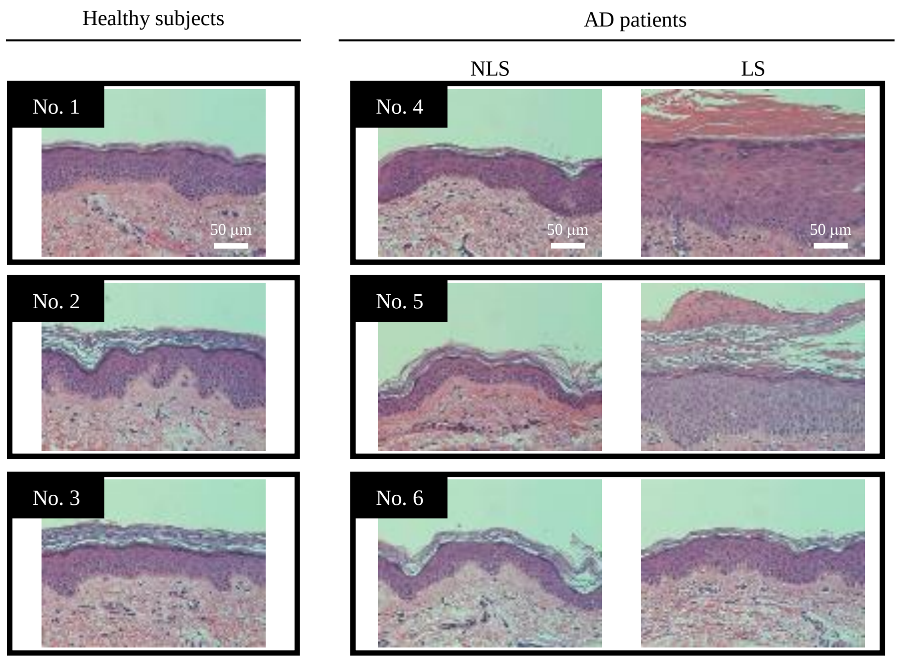

Supplement: S1 Table — (PDF) [file pone.0161759.s003.pdf]
