# Supplementary material for: Impaired Tight Junctions in Atopic Dermatitis Skin and in a Skin-Equivalent Model Treated with Interleukin-17
Source: PLoS One. 2016 Sep 2;11(9):e0161759. doi: 10.1371/journal.pone.0161759 (PMC5010286; doi:10.1371/journal.pone.0161759)
Supplement: S2 Table — (PDF) [file pone.0161759.s004.pdf]

S2 Table The details of numeric data

	Fig.2a				Fig.5a	Fig.5c
	TER value (Ω･cm^2)				profilaggrin/GAPDH (Arbitrary Unit)	Amino Acid (nmol/ φ8mm)
cytokine	TNF-α	IL-4	IL-17	1L-22	IL-17	IL-17
Number of samples	6	6	6	6	3	4
0 ng/ml	1165	1066	1117	995	1.0	95.1
	808	1010	1167	910	1.0	111.6
	1099	866	1088	907	1.0	100.6
	1149	1052	1126	993	-	105.2
	801	980	1197	909	-	-
	1097	829	1131	909	-	-
AV	1020	967	1138	937	1.0	103.1
STDEV.P	154	90	35	40	0.0	6.1
1 ng/ml	993	932	1016	1014	1.2	113.3
	1033	988	813	932	1.1	99.2
	1058	998	972	971	0.9	101.2
	987	937	1040	1032	-	105.5
	1051	995	840	935	-	-
	1083	1015	1004	972	-	-
AV	1034	978	948	976	1.1	104.8
STDEV.P	35	32	89	37	0.1	5.4
p value (t-test, vs 0 ng/ml)	0.84	0.81	0.00	0.14	0.42	0.73
10 ng/ml	751	1057	871	1101	1.0	77.9
	1141	991	925	1002	0.9	102.8
	1088	931	902	1071	0.8	103.0
	761	1059	895	1102	-	93.4
	1150	1005	944	1003	-	-
	1105	942	920	1075	-	-
AV	999	997	910	1059	0.9	94.3
STDEV.P	173	50	24	42	0.1	10.2
p value (t-test, vs 0 ng/ml)	0.85	0.52	0.00	0.00	0.11	0.24
100 ng/ml	1135	862	903	988	0.9	99.0
	905	1016	999	1131	0.9	77.1
	1012	868	903	998	0.7	81.4
	1139	856	942	987	-	79.4
	924	1028	1027	1126	-	-
	1037	884	943	1001	-	-
AV	1025	919	953	1038	0.8	84.2
STDEV.P	91	74	46	64	0.1	8.7
p value (t-test, vs 0 ng/ml)	0.95	0.37	0.00	0.01	0.09	0.02
